# Switching diet from Egyptian pyramids to Amazonian fruits: changing our menu using epidemiological evidence

**DOI:** 10.1590/S1516-31802002000100001

**Published:** 2002-01-02

The pyramids were built in Ancient Egypt. Nowadays, pyramids provide us with the most perfect icon to associate with healthy eating. The foods that can be eaten every day are usually presented at the bottom of the pyramid and those we have to eat sparingly are at the top.

The most famous food pyramid comes from the United States Department of Agriculture, as a guide for healthy nutrition. Epidemiological evidence has been showing that the United States Department of Agriculture's pyramid is not good for the Americans and so it will probably also not be good for Brazilians. This pyramid (yes, it is not a recommendation made by human beings, but a mystical order from a mystical ancient symbol!) puts carbohydrates like bread, cereal, rice and pasta at the bottom. Fruits and vegetables form the second level. The third level consists of meat, poultry, fish, dried beans, eggs and nuts, and also the dairy products milk, yogurt and cheese. Fats, oils and sweets make up the highest level (the ones we need to eat sparingly).^1^

This nutritional recommendation has transformed the United States into a fat-free society, but not a society without fat people! The Americans have taken on board the pyramid's message: you can eat a lot of pasta, bread, rice and potatoes but you cannot eat fat! In the 1960s Americans were obtaining 40% of their calories from fats, but during the 1990s this proportion fell to 34%.^2^ And, if this is true, how come the Americans are the fattest people in the world? The answer is in the switch from fats to carbohydrates. The replacement of fat-based energy by carbohydrate energy increases body weight and decreases the total cholesterol level but it also reduces the HDL-cholesterol, and increases triglycerides. Under these conditions, the lipid profile is changed, but to a worse shape!

However, the most important point is to refute the claims of scientific justifications for blaming either fat-rich foods or oils. Not all fats are unhealthy. Saturated fats from diary products and *trans*-fat from margarine are very bad for the atherosclerotic process. However, vegetable shortening (highly hydrogenated fats from vegetables, which are mostly *trans*-fatty) is more dangerous than saturated fats. *Trans*-fats are formed when vegetable oils are hydrogenated to form solid fats like margarine. However, other types of fats like polyunsaturated and monounsaturated fats found in olive oils, nuts, whole grains and fish are very good for our hearts and our health. So, the solution is not to banish fats from our diets but to change the fats that we eat.^3^

Not all the complex carbohydrates are good. Rice, potatoes, bread and pasta are rapidly transformed into sugar after digestion. They have a high glycemic index, promoting rapid increase in insulin levels. But after digestion, you will be hungry again very soon. So, what we have to do, is to substitute for these complex carbohydrates via whole-grain carbohydrates, like those in whole-wheat pasta or bread or in beans. However, recent studies in Brazil have shown a decline in the consumption of beans, fruits and vegetables and a further increase in simple sugar consumption.^4^

Protein is very important in a diet. However, it is good if it comes from fish and poultry and not from red meat. Fish delivers some important unsaturated fats as well. However, vegetal proteins (as in beans and nuts) are also very good and they have some advantages over animal sources of protein. They give us fiber, vitamins, and minerals. Most epidemiological studies have been showing that increased fruit and vegetable intake is directly associated with lowered incidence of cardiovascular disease. Moreover, fruits and vegetables are a source of calcium, and we can ingest a lot of calcium from vegetables with dark leaves such as broccoli, spinach and all the brassica family.

One traditional enemy must be reconsidered. Eggs are a very important source of essential fats and two cohort studies using good nutritional assessment data have not shown any association with cardiovascular diseases. On the other hand, the traditional friend margarine has gone. It used to be very common to hear statements like "margarine is better than butter". However, if you eat a lot of margarine rich in *trans-*fat, this is worse than butter with saturated fat.

Nonetheless, the most important lesson that come from epidemiological studies is that if you eat a lot of fruits, whole grains and "good" fat, then you become overweight. And weight gain are riskier for health than some specific kinds of "unhealthy foods". As Walter Willett and Patrick Skerrett stated in their book, the most important thing is to maintain our weight: the weight we had in early adulthood. This is the best way to prevent a lot of chronic diseases.^5^

Brazil is not so different from the United States nowadays. Three comparable household surveys, undertaken in two of the most highly populated Brazilian regions in 1975 (n = 95,062), 1989 (n = 15,580) and 1997 (n = 10,680), provided annualized anthropometric measurements and socioeconomic data that showed an increasing epidemic of obesity. This is now most severe among men, in rural settings and among poorer people.^6^ Weight gain is a risk factor for hypertension, dyslipidemia and diabetes, three of the most important cardiovascular risk factors. From these data, it will be reasonable to expect a new epidemic of cardiovascular disease in the next few years.^7^

What is the solution for this problem? What would be good proposals for the Brazilian diet? The key to solving the diet-disease dilemma is to be found by reading an interview given by the famous Brazilian parasitologist, Luiz Hildebrando Pereira da Silva. He described how children living in small towns in Rondônia were able to pick almost 50 different kinds of fruit, but in contrast, the local researchers did not have enough tools and skills to identify all of them.^8^ This serves to demonstrate our incredible biodiversity. We need to test the Brazilian diet using advanced epidemiological tools, considering some important endpoints such as all-cause mortality, cardiovascular and cancer mortality and incidence. This procedure will be good for Brazilians and for the Brazilian economy and needs to be considered as a strategic policy for our Ministry of Science and Technology in relation to diet and nutrition.

Concluding, we have to seek a healthy diet by disregarding erroneous models such as the American one, which paradoxically has given rise to the world's most obese society through the consumption of fat-free foods. The best advice regarding diets for all is to leave the pyramids alone, for Egyptologists and tourists to enjoy.
